# Transmission of *Leishmania* metacyclic promastigotes by phlebotomine sand flies

**DOI:** 10.1016/j.ijpara.2007.04.003

**Published:** 2007-08

**Authors:** Paul A. Bates

**Affiliations:** Liverpool School of Tropical Medicine, Pembroke Place, Liverpool L3 5QA, UK

**Keywords:** *Leishmania*, *Viannia*, Life cycle, Promastigote secretory gel, Metacyclic promastigotes, Regurgitation, Sand fly, Saliva

## Abstract

A thorough understanding of the transmission mechanism of any infectious agent is crucial to implementing an effective intervention strategy. Here, our current understanding of the mechanisms that *Leishmania* parasites use to ensure their transmission from sand fly vectors by bite is reviewed. The most important mechanism is the creation of a “blocked fly” resulting from the secretion of promastigote secretory gel (PSG) by the parasites in the anterior midgut. This forces the sand fly to regurgitate PSG before it can bloodfeed, thereby depositing both PSG and infective metacyclic promastigotes in the skin of a mammalian host. Other possible factors in transmission are considered: damage to the stomodeal valve; occurrence of parasites in the salivary glands; and excretion of parasites from the anus of infected sand flies. Differences in the transmission mechanisms employed by parasites in the three subgenera, *Leishmania*, *Viannia* and *Sauroleishmania* are also addressed.

## 1 Introduction

The genus *Leishmania* are parasitic protozoa responsible for the leishmaniases, a group of diseases affecting human and various animal populations throughout much of the tropics and subtropics. Over 30 species of *Leishmania* have been named to date, and of these 10 or so are of significant medical and veterinary importance (Bates and Ashford, 2006; Lainson and Shaw, 2006). The major clinical syndromes found in human beings are cutaneous, mucocutaneous and visceral leishmaniasis, but these can present in a wide variety of forms. The only proven route of infection is by the bite of female phlebotomine sand flies (Bates and Rogers, 2004). Phlebotomine sand flies are dipteran insects within the family Psychodidae and approximately 700 species have been described to date. Of these ∼10% have been incriminated as vectors of leishmaniasis with varying degrees of certainty; convincing evidence of vectorial capacity has been demonstrated for about 30 species (Lane, 1993). Some of the most important vector–parasite combinations and the resulting diseases are summarised in Table 1. The transmission mechanisms used by *Leishmania* are key to the maintenance of the life cycle and their importance as disease-causing organisms. For the purposes of this review, transmission is defined as those events surrounding the inoculation of parasites into the vertebrate host.

## 2 Development in the vector

**Fig. 1:**
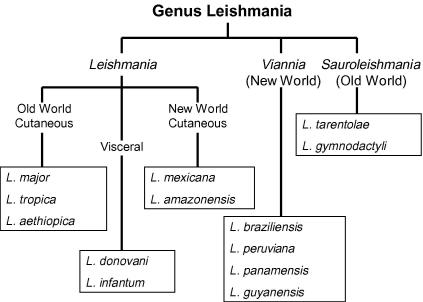
Outline classification of *Leishmania* illustrating the three subgenera. The list of named species is not comprehensive; over 30 species have been named in the genus including many that are non-pathogenic or of minor medical importance (of limited range or small numbers of recorded cases). The species named above include some of the better known species that are the focus of biomedical research. Parasites in the subgenera *Leishmania* and *Viannia* infect mammals, whereas the *Sauroleishmania* infect reptiles as their vertebrate hosts.

Currently there are three groups of *Leishmania* parasites that are classified into different subgenera (Fig. 1) and these vary depending on which parts of the sand fly gut are colonised by the parasites. Indeed, the original division of the mammal-infective leishmaniae into subgenera *Leishmania* and *Viannia* by Lainson and Shaw was largely based on this character (Lainson et al., 1977), a separation which has been subsequently supported by DNA sequence-based phylogenetic analyses (Croan et al., 1997; Noyes et al., 2002). The following account of developmental biology is largely based on the subgenus *Leishmania* (*Leishmania*), for which the most reliable and complete information is available. Differences in the subgenus *Leishmania* (*Viannia*) are then described, and finally the *Leishmania* (*Sauroleishmania*) are discussed, about which still relatively little is known.

### 2.1 Leishmania (Leishmania)

**Fig. 2:**
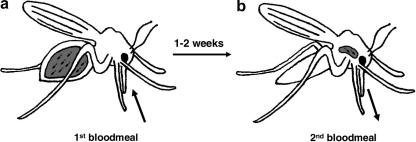
Bloodfeeding and transmission of *Leishmania*. (a) Sand flies become infected when amastigotes are ingested along with a bloodmeal and transform into promastigotes. During the “bloodmeal phase” the parasites are located at the posterior end of the midgut, and such infections are immature and non-transmissible. (b) Development in the gut continues for 1–2 weeks resulting in a mature transmissible infection with metacyclic promastigotes located in the anterior of the gut. Depending on parasite and vector species there may be additional bloodmeals during the maturation period, but most parasites can complete their development within the timeframe of a single digestive cycle. During maturation the flies supplement their nutrition by feeding on sugars from plants, so this is sometimes known as the “sugarmeal phase” of parasite development. Infective metacyclic promastigotes are egested when the fly takes a subsequent bloodmeal.

**Fig. 3:**
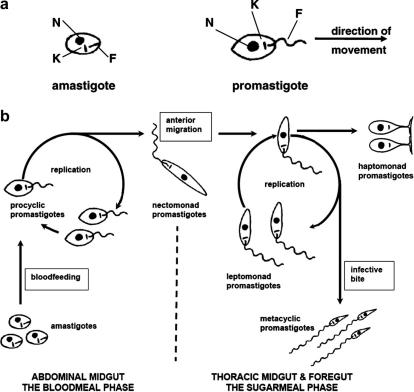
Development of *Leishmania* (*Leishmania*) species in the sand fly vector. (a) The morphology of amastigotes and promastigotes. Each form has a nucleus (N), kinetoplast (K) and flagellum (F). The kinetoplast is the mitochondrial genome. The flagellum in amastigotes is internal and non-functional; in promastigotes the flagellum extends from the cell body, beats and pulls the organism in the direction shown, emerging from the anterior end of the cell. (b) The developmental sequence of the five major promastigote forms: procyclic promastigotes, nectomonad promastigotes, leptomonad promastigotes, haptomonad promastigotes and metacyclic promastigotes. The exact position of haptomonad promastigotes in the developmental sequence is uncertain.

Female sand flies (*Phlebotomus* species in the Old World, *Lutzomyia* species in the New World) acquire *Leishmania* parasites when they feed on an infected mammalian host in search of a bloodmeal (Fig. 2). The parasites, amastigote forms, that are taken up by sand flies are not usually found in the peripheral circulation, rather they are present in the skin itself. Parasites present in organs such as liver and spleen are not accessible to sand flies. Amastigotes are intracellular parasites found in phagolysosomes of macrophages and other phagocytes (Handman and Bullen, 2002), and their uptake by the bloodfeeding sand fly is assisted by the cutting action of the mouthparts. Thus sand flies are pool feeders, meaning they insert their saw-like mouthparts into the skin, and agitate them to produce a small wound into which the blood flows from superficial capillaries (Lane, 1993). It is this tissue damage associated with the creation of the wound that releases skin macrophages and/or freed amastigotes into the pool of blood, and enables their subsequent uptake into the abdomen of the sand fly. The change in conditions moving from the mammalian host to the sand fly midgut (decrease in temperature, increase in pH) triggers development of the parasite in the vector (Bates and Rogers, 2004; Kamhawi, 2006). The amastigotes transform into motile promastigotes with flagella beating at the anterior end (Fig. 3a). This first stage in the vector is called a procyclic promastigote – it is a weakly motile, replicative form that multiplies in the bloodmeal (Fig. 3b). This initial “bloodmeal phase” is confined by the peritrophic matrix, a chitin and protein mesh secreted by the midgut epithelium that encloses the blood being digested within (Secundino et al., 2005). After a few days, the parasites begin to slow their replication and differentiate into elongate, strongly motile nectomonad promastigotes. These are migratory forms that accumulate at the anterior end of the peritrophic matrix and break out of the bloodmeal. This escape is facilitated by the action of a parasite secretory chitinase (Schlein et al., 1991; Shakarian and Dwyer, 2000) and probably by the action of endogenous sand fly chitinase (Ramalho-Ortigao et al., 2005). They move towards the anterior midgut, some of them attaching to the microvilli of the midgut epithelium, until they reach the stomodeal valve (cardia) that guards the junction between foregut and midgut. These nectomonad promastigotes mediate the establishment phase of the infection that marks a true vector i.e. persistence beyond the bloodmeal and avoidance of expulsion during defecation. Thus the ability to attach is an important property of *Leishmania* (*Leishmania*) promastigotes (Sacks and Kamhawi, 2001). The major parasite surface glycoconjugate lipophosphoglycan (LPG) is responsible for binding to a galectin on the sand fly gut epithelium in certain species e.g. *Leishmania major* in *Phlebotomus papatasi* (Pimenta et al., 1992; Kamhawi et al., 2004), although recent findings indicate that non-LPG mediated attachment is used by some other *Leishmania* species (Rogers et al., 2004; Svobodova et al., 2006; Volf and Myskova, 2007; Myskova et al., 2007). The identity of these alternative receptor–ligand pairs has not been fully described. Once they reach the stomodeal valve the nectomonad promastigotes transform into leptomonad promastigotes, shorter forms that resume replication (Gossage et al., 2003). These are responsible for the secretion of promastigote secretory gel (PSG; Rogers et al., 2002), which plays a key role in transmission as described below. Some of the nectomonad/leptomonad promastigotes also attach themselves to the cuticle-lined surface of the valve and differentiate into haptomonad promastigotes (Killick-Kendrick et al., 1974). This form of attachment is mechanistically different to that seen with the midgut epithelium and is mediated by expansion of the flagellar tip into hemi-desmosome-like structures (Vickerman and Tetley, 1990; Wakid and Bates, 2004). Finally, some of the leptomonads differentiate into metacyclic promastigotes (Rogers et al., 2002), which are the mammal-infective stages. These are deposited in the skin of a new mammalian host when the fly takes another bloodmeal, leading to the transmission of disease.

### 2.2 Leishmania (Viannia)

These parasites are only found in the New World and therefore all the vectors are *Lutzomyia* species. The initial events for these parasites in their vectors are similar to those described above for the subgenus *Leishmania*. Amastigotes are taken up and transform into procyclic promastigotes, which replicate in the bloodmeal. Following this, however, the majority of the parasites can be found in the pyloric region of the hindgut (Walters et al., 1989; Nieves and Pimenta, 2000), a defining feature of the subgenus. Few studies have been made where quantitation has been performed, but a minor population probably also goes forward to the anterior midgut at the same time as the major hindgut migration. Given this, it is possible that the hindgut migration is not an essential part of the life cycle, and is not required for the development of transmissible infections. However, the most likely scenario, based on current evidence, is that the backward migration and hindgut development are integral parts of the life cycle. The role of the hindgut phase is supported by the observation that the parasites firmly attach themselves as haptomonad promastigotes to the cuticle-lined hindgut (Walters et al., 1989). Conversely, there is relatively little evidence for a midgut epithelial attachment phase in the *Viannia* (Molyneux and Killick-Kendrick, 1987; Soares et al., 2005). Thus, one possible explanation for the hindgut phase is that it serves the function of establishment in the vector for these parasites. After the hindgut phase there is a forward migration of parasites, followed by accumulation in the anterior midgut, secretion of PSG (Gontijo and Bates, unpublished observations) and differentiation of metacyclic promastigotes. Again the detailed kinetics of this anterior migration have not been fully explored. However, although the route may be slightly different and the parasite stages involved have not been defined, it appears that the end result of development in the *Viannia* is similar to that in the subgenus *Leishmania*.

### 2.3 Leishmania (Sauroleishmania)

As the name suggests this group of species are the “lizard *Leishmania*”. They are non-pathogenic to humans, and *Leishmania tarentolae* in particular has become a popular model organism for that reason. They have been included or excluded from the genus at various points in their history, sometimes regarded as a “primitive” group, latter day representatives of ancestral *Leishmania* species. However, recent DNA sequence-based phylogenies have clearly placed them within the genus (Croan et al., 1997; Noyes et al., 1997; Orlando et al., 2002), indicating that they are a secondarily derived development from the mammalian species, rather than representing some ancient group. Unfortunately, little work has been done regarding development in their vectors. Their development has been described as hypopylarian, meaning that it is confined to the hindgut, although there have been a few claims of anterior midgut development in the older literature (Wilson and Southgate, 1979; Zhang and Leng, 1997). The vectors are members of the genus *Sergentomyia*, sand flies that are known to feed on reptiles as well as other vertebrates. *Sergentomyia* species are widely distributed throughout the Old World. There are very few reports of the development of these parasites in their vectors and transmission has never been experimentally demonstrated. The parasites are taken up during bloodfeeding and, although not described, this probably occurs via a pool feeding mechanism as for the mammalian vectors. However, the associated tissue damage is not required for *Sergentomyia* to acquire *Leishmania* (*Sauroleishmania*) parasites, since in their reptile hosts the *Sauroleishmania* are found in the circulation as extracellular promastigotes or amastigotes in monocyte-like cells or erythrocytes (Wilson and Southgate, 1979; Paperna et al., 2001). Once in their sand fly hosts the bloodmeal is enclosed in a peritrophic matrix and multiplication of the parasites as promastigotes occurs. However, *Sergentomyia* species produce a relatively thick peritrophic matrix compared with *Phlebotomus* and *Lutzomyia*, which may be part of the reason why development in the anterior midgut is not favoured or not possible (Lawyer et al., 1990; Shatova et al., 1991). Perhaps the parasites are passed into the hindgut because they cannot escape from the peritrophic matrix early enough in response to the rate of bloodmeal digestion and defacation. This seems to be part of the reason why *Sergentomyia schwetzi* was an unsuitable experimental vector for *L. major* (Lawyer et al., 1990). It is not known what further developmental stages occur in the *Sauroleishmania*, and specifically whether metacyclic promastigotes are produced. However, it is reasonable to assume that a reptile-infective form is produced, by analogy with the mammal-infective species of *Leishmania*.

## 3 Transmission mechanisms

### 3.1 Inoculation versus regurgitation

The development of infections in the anterior midgut/foregut, and demonstration that phlebotomine sand flies could transmit *Leishmania* by bite, were key advances made by Saul Adler, Oscar Theodor, Henry Short and others in the early 20th century (Molyneux and Killick-Kendrick, 1987). However, from the beginning there was debate about the mechanism of transmission, with two schools of thought advocated by various workers at different times. Short advocated the idea of regurgitation, something analogous to what had been proposed earlier for the plague bacillus in fleas (Bacot and Martin, 1914). It was proposed that promastigotes in the gut formed a physical obstruction that had to be removed by regurgitation, an idea that came to be known as the “blocked fly hypothesis”. Others favoured inoculation, by which they meant that only promastigotes found in the proboscis were involved in transmission, these forms being introduced into the skin during biting. According to this idea, the occurrence of “proboscis forms” was required for a particular sand fly to act as a vector. This theory was based on the observation that flies could be seen to “probe” the skin several times i.e. insert their mouthparts for short periods of time, fail to take a bloodmeal or only a partial meal, and yet lesions would still develop (Killick-Kendrick et al., 1977; Beach et al., 1985). This idea gained further currency with observations such as a single infected fly probing repeatedly and generating 11 lesions on the arm of a volunteer (Beach et al., 1984). However, an underlying assumption of the traditional inoculation theory is that such probing is a relatively passive process and is not accompanied by regurgitation.

### 3.2 Chitinase damage to stomodeal valve

The occurrence of regurgitation during natural transmission has been suggested by several authors (Jefferies et al., 1986; Killick-Kendrick, 1986; Warburg and Schlein, 1986). A more recent variation on these theories was advocated by Schlein and colleagues who observed that the cuticle-lined stomodeal valve became damaged in infected sand flies (Schlein et al., 1992), and proposed that the parasite secretory chitinase was responsible for this, since chitin is a major constituent of cuticle. They partially resurrected the regurgitation theory, arguing that a damaged and, therefore, partially non-functional valve facilitated the “reflux” of parasites from the midgut via the sequential action of pharyngeal and cibarial pumps during bloodfeeding. Evidence for damage to the stomodeal valve has since been provided by another study (Volf et al., 2004), so it seems that such damage is a feature of at least some vector–parasite combinations. However, it is still unclear whether this is an essential part of the transmission mechanism, i.e. without damage transmission cannot occur or, perhaps more likely, such damage is a pathogenic by-product of infection that may facilitate transmission under some circumstances. This question requires further experimental investigation.

### 3.3 Promastigote secretory gel

**Fig. 4:**
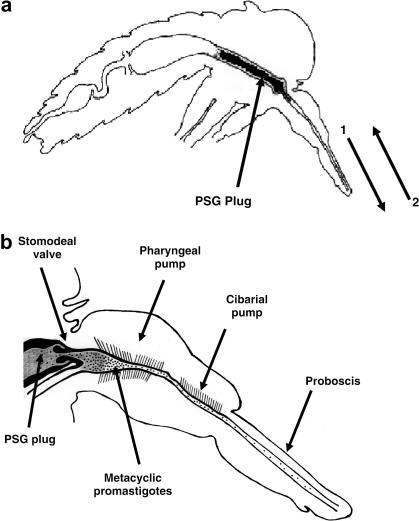
(a) Sagittal section through a *Leishmania*-infected female sand fly showing the position of the promastigote secretory gel (PSG) plug within the anterior midgut and foregut. The plug must be partially egested (1) before blood feeding can occur (2), thereby injecting both metacyclic promastigotes and PSG into the skin of the mammalian host. (b) Detail of the anterior midgut and foregut. The PSG plug (shaded) forces the stomodeal valve open and extends into the pharynx region. Metacyclic promastigotes (stippling) are concentrated at the anterior pole of the plug but are found along the foregut in both the cibarium and proboscis.

Recent work has helped to resolve some of these differences in opinion, and can incorporate all of the previous data. The first key finding was the observation that a hitherto mysterious gel-like substance that had been noted in infected sand flies by many workers was actually a parasite product (Stierhof et al., 1999), something we have named PSG (Rogers et al., 2002). The main component of PSG is a high molecular weight glycoprotein called filamentous proteophosphoglycan (fPPG; Ilg et al., 1996). The identification of PSG gave new credence to the blocked fly hypothesis, because the gel-forming properties of fPPG can provide the required physical obstruction. In situ, PSG forms into a sausage-like form (Rogers et al., 2002), filling and distending the anterior midgut of the sand fly, extending through the stomodeal valve into the foregut (Fig. 4). Although fPPG is clearly the major component of PSG, and the critical component for transmission and disease exacerbation (see below), other secretory products of the parasite or the sand fly may be present in the plug that could have as yet undescribed biological effects. It should also be noted that the PSG plug obstructing the anterior midgut is packed with promastigote cell bodies. Further contributing to the blockage of the gut are the haptomonad promastigotes, attached to the cuticular lining of the stomodeal valve and the foregut. Whether they are essential for transmission is unclear, probably not, but they may facilitate the generation of the PSG plug by providing an initial scaffold of cell bodies. The position of the haptomonad promastigotes in the developmental cycle is uncertain, but they may represent a terminally differentiated stage. For this reason they have been termed “altruistic” forms, since they cannot be transmitted themselves but aid the transmission of their genetically identical siblings, the metacyclic promastigotes. Essentially the plug is like a cell pellet of promastigotes embedded in PSG. When a PSG plug is observed in situ by microscopy there is little evidence of life within, the cells appear immobile. However, when dissected out into culture medium the PSG readily dissolves, and the freed promastigotes regain their motility. The majority of these are leptomonad promastigotes, one of the main pieces of evidence that this life cycle stage is responsible for the secretion of PSG. The other life cycle stages associated with the plug are metacyclic promastigotes. Interestingly these are mainly located at the poles of the plug, in an ideal position for transmission. Although some metacyclic promastigotes are found in the proboscis, the majority were associated with the PSG plug.

### 3.4 Egestion and disease exacerbation in the mammalian host

The work described above is strong support for the blocked fly hypothesis, and by inference indirect support for the regurgitation theory of transmission. Direct proof of regurgitation was provided by further work, where the numbers of parasites egested by infected sand flies were compared with those present in the foregut of such flies (Rogers et al., 2004). The numbers egested were approximately 10-fold greater than in the foregut, and could only be explained if active regurgitation accompanied transmission by fly bite. This study also demonstrated that PSG was egested along with metacyclic promastigotes during transmission – this makes sense, since the sand fly must clear a way, expelling at least some of the PSG from the foregut/midgut to make a channel through which blood can then be imbibed, something we have directly observed (Rogers and Bates, unpublished observations). This will have the inevitable consequence, highly desirable for the parasite, of simultaneously expelling metacyclic promastigotes into the skin of the waiting mammalian host. The observation that PSG is egested during transmission means that there are now three known components of the infective inoculum: the metacyclic promastigotes themselves, which are obviously essential for transmission; sand fly saliva; and PSG. Sand fly saliva is a well established disease exacerbation factor (Titus and Ribeiro, 1988), at least for cutaneous leishmaniasis. It is required for blood feeding activity, having potent vasodilatory and antihaemostatic properties (Ribeiro, 1987). Co-inoculation of sand fly saliva with parasites has been shown to result in disease exacerbation in several studies, and this appears to be due to modulation of the immune response to favour parasite survival and replication (Kamhawi, 2000; Rohousova and Volf, 2006). Similarly, PSG has also been shown to possess disease exacerbation properties, leading to an increase in pathology and parasite numbers when co-inoculated with metacyclic promastigotes (Rogers et al., 2004). The existence of these two sources of disease exacerbation factors leads to the question, which is the most important of the two? There is probably no single answer to this question, the actual amounts of saliva and PSG egested being likely to vary between parasite and vector combinations, or even between individual sand flies, since no two infections or fly bites will be identical. In our study, we directly compared the two and showed that PSG was a much more potent disease exacerbation factor (Rogers et al., 2004). If generally true, this is probably a reflection of selection acting directly on a parasite product compared with acting indirectly on the vector to supply an important component of the infective inoculum, since it is more difficult to see how natural selection would act to maximise transmission in the latter situation. However, it could certainly be true that egestion of suitable amounts or quality of saliva is an important component of vector specificity – only certain sand fly species being equipped to function as vectors. On the other hand, the production of a parasite product (PSG) that enhances the transmission of the parasite itself is clearly of direct advantage, and would be subject to very strong selective pressure, both in terms of quantity (to produce high amounts of PSG) and quality (for optimal mechanical and immunological properties) of the material secreted. Nevertheless, one important caveat applies to all this work on saliva and PSG as exacerbation factors: none of the published studies have been performed with natural hosts i.e. the relevant wild rodent and other hosts listed in Table 1. Since this is where natural selection will act, studies using these hosts are required before we can fully understand the relative importance of the various components of the infective inoculum.

### 3.5 Transmission in subgenus Viannia

As described above, although the *Viannia* parasites have a hindgut phase in their development, transmission still occurs by the anterior route. There has been little work on transmission with these parasites, especially using the sand fly vectors. To my knowledge experimental transmission of *Viannia* parasites by infected sand fly bite has not been achieved to date. However, *Leishmania braziliensis* does produce PSG in *Lutzomyia longipalpis* (Gontijo and Bates, unpublished observations) and PSG-like material has been noted in *Leishmania panamensis*-infected sand flies (Walters et al., 1989). Thus the current evidence is suggestive that a similar mechanism will apply to these parasites as described above for the subgenus *Leishmania*. This awaits experimental verification.

### 3.6 Salivary glands

The presence of parasites in the salivary glands of sand flies has been reported by some workers and this has been proposed to be of relevance to transmission (Killick-Kendrick et al., 1996; Freitas et al., 2002). This is an attractive idea, given that saliva must be egested into the wound to assist blood feeding, and the observations described above that saliva helps to exacerbate cutaneous leishmaniasis. The route by which the parasites reached the glands is uncertain – the two possibilities being travelling down the proboscis and back up the salivary duct (analogous to African trypanosomes) or through the midgut wall, haemocoel and penetrating the gland wall (analogous to malaria ookinetes and sporozoites). However, to date these findings have not been substantiated by others, and the low reported frequency of infected glands together with the difficulty of dissecting out the salivary glands without inadvertent contamination from parasites in the gut (which is usually severed during dissection) makes it difficult to assess the significance of these findings. At present there is no convincing evidence to support the notion that this is a normal route of transmission.

### 3.7 Excretion via the posterior station

Another interesting observation is that microcapillary feeding of infected sand flies can lead to the appearance of parasites in the “urine” emitted from the anus (Sadlova and Volf, 1999). This is a somewhat artificial scenario, however, it has been also noted that (uninfected) sand flies do undergo pre-diuresis when bloodfeeding under normal conditions. This has been observed in both *Phlebotomus* (Sadlova et al., 1999) and *Lutzomyia* (Dillon, personal communication). Pre-diuresis is the concentration of the cellular component of the blood during feeding, the excess plasma being voided in droplets excreted from the anus. Thus, although it has not been demonstrated, it is possible that parasites could be excreted from the posterior station in infected flies feeding normally on mammalian hosts. However, such parasites are likely to be non-infectious whereas metacyclic promastigotes are found in an anterior position. Further, the feeding difficulties of infected sand flies described above would seem to make this a remote possibility under natural conditions. Therefore, at present this route of transmission does not seem to be significant.

### 3.8 Sauroleishmania

Little, if anything, is known about transmission of the *Sauroleishmania*. Given their confinement to the hindgut it is possible that they are transmitted in a similar fashion to *Trypanosoma cruzi* i.e. defecated during blood feeding and then introduced inadvertently into the wound, broken skin or mucosal surfaces by rubbing the skin. However, unlike triatomine bugs, sand flies generally defecate a few days before bloodfeeding again and have a “sugarmeal phase” before seeking out another vertebrate host. It is possible that prediuresis may facilitate transmission of these parasites as described above. However, the general assumption is that transmission is achieved by the reptile host eating an infected sand fly (Wilson and Southgate, 1979). The parasites could then gain access to the circulation through the mucosal surfaces of the reptile gut. This route of transmission seems plausible, but remains to be experimentally demonstrated. Whatever the route, it must be effective enough to maintain the life cycles of these parasites in nature.

## 4 Unanswered questions and future directions

As this review has demonstrated, there has been significant recent progress in understanding the transmission mechanism of *Leishmania*. However, large gaps in our knowledge still remain, and from the medical perspective the most urgent of these is to determine the transmission mechanism of the subgenus *Viannia* species. For example, an understanding of transmission in this group of parasites may help to explain the onset of destructive mucocutaneous disease caused by *L. braziliensis*. Another important area of future research is the possible development of anti-saliva vaccines (Kamhawi et al., 2000; Valenzuela et al., 2001; Oliveira et al., 2006). Whether saliva itself plays a critical role in parasite transmission or not, it is certainly essential for blood feeding and anti-saliva immune responses can interfere with transmission. Therefore, sand fly salivary antigens may become components of future *Leishmania* vaccines. With respect to vaccines, perhaps the most important future application of our improved understanding of transmission is in the evaluation of vaccine candidates. It is clear that natural transmission by bite, inclusion of PSG and/or saliva, or even the usage of metacyclic promastigotes in appropriate numbers are rarely used in models of experimental leishmaniasis. There are reasons for this of course – these are not materials readily to hand in many laboratories. However, they are likely to be crucial for the development of effective vaccines against leishmaniasis (Rogers et al., 2006).

## Figures and Tables

**Table 1:** Medically important species of phlebotomine sand fly and transmission of leishmaniasis

Sand fly species	Geographical distribution	Species of *Leishmania*	Main disease(s) in humans	Transmission	Important mammalian hosts
*Phlebotomus papatasi*, *Phlebotomus dubosqi*, *Phlebotomus salehi*	Central and West Asia, North Africa, Sahel of Africa, Central and West Africa	*Leishmania (Leishmania) major*	Cutaneous (oriental sore)	Rural zoonotic	Great gerbil (*Rhombomys opimus*), fat sand rat (*Psammomys obesus*)
					
*Phlebotomus sergenti*	Central and West Asia, North Africa	*Leishmania (Leishmania) tropica*	Cutaneous (oriental sore)	Urban anthroponotic	Humans, rock hyraxes
					
*Phlebotomus longipes*, *Phlebotomus pedifer*	Ethiopia, Kenya	*Leishmania (Leishmania) aethiopica*	Cutaneous diffuse cutaneous	Rural zoonotic	Rock hyraxes (*Heterohyrax brucei*, *Procavia* spp.)
					
*Phlebotomus argentipes*, *Phlebotomus orientalis*, *Phlebotomus martini*	Indian subcontinent, East Africa	*Leishmania (Leishmania) donovani*	Visceral (kala azar)	Epidemic anthroponotic	Humans
					
*Phlebotomus ariasi*, *Phlebotomus perniciosus*	Mediterranean basin, Central and West Asia	*Leishmania (Leishmania) infantum*	Infantile visceral	Zoonotic peridomestic	Domestic dog
					
*Lutzomyia longipalpis*	Central and South America	*L. (L.) infantum (syn. chagasi)*	Infantile visceral	Zoonotic peridomestic	Domestic dog, foxes (*Lycalopex vetulus*, *Cerdocyon thous*)
					
*Lutzomyia olmeca olmeca*	Central America	*Leishmania (Leishmania) mexicana*	Cutaneous (chiclero’s ulcer)	Sylvatic zoonotic	Forest rodents (*Ototylomys phyllotis* + others)
					
*Lutzomyia flaviscutellata*	South America	*Leishmania (Leishmania) amazonensis*	Cutaneous	Sylvatic zoonotic	Forest rodents (*Proechimys* spp. + others)
					
*Lutzomyia wellcomei*, *Lutzomyia complexus*, *Lutzomyia carrerai*	Central and South America	*Leishmania (Viannia) braziliensis*	Cutaneous mucocutaneous (espundia)	Sylvatic zoonotic	Forest rodents (*Akodon* spp., *Proechimys* spp. + others)
					
*Lutzomyia peruensis*, *Lutzomyia verrucarum*	Peru	*Leishmania (Viannia) peruviana*	Cutaneous (uta)	Upland zoonotic	Reservoir unknown, dog?
					
*Lutzomyia umbratilis*	South America	*Leishmania (Viannia) guyanensis*	Cutaneous, often metastatic (pian-bois)	Sylvatic zoonotic	Sloth (*Choloepus didactylus*), anteater (*Tamandua tetradactyla*)
					
*Lutzomyia trapidoi*	Central America	*Leishmania (Viannia) panamensis*	Cutaneous	Sylvatic zoonotic	Sloth (*Choloepus hoffmanni*)

Various species in the genus *Phlebotomus* are responsible for transmission of leishmaniasis in the Old World and *Lutzomyia* species in the New World. Each sand fly species typically transmits only one species of parasite and each parasite leads to a particular type of disease. Animal reservoirs are important for maintaining the life cycle of many *Leishmania* species and consequently transmission is frequently zoonotic and rural/sylvatic. Important exceptions are *Leishmania tropica* and *Leishmania donovani*, which are transmitted between human beings.
